# The association of erythrocyte sedimentation rate, high-sensitivity C-reactive protein and diabetic kidney disease in patients with type 2 diabetes

**DOI:** 10.1186/s12902-020-00584-7

**Published:** 2020-07-13

**Authors:** Shizhe Guo, Meng Wang, Yifei Yu, Yeping Yang, Fangfang Zeng, Fei Sun, Qin Li, Min He, Yiming Li, Jie Wen, Wei Gong, Zhaoyun Zhang

**Affiliations:** 1 Department of Endocrinology and Metabolism, Huashan Hospital, Fudan University, 12 Wulumuqi Road, Shanghai, 200040 China; 2 Department of Endocrinology, Shanghai Ninth People’s Hospital, Shanghai Jiaotong University, Shanghai, China; 3 Department of Endocrinology, Jing’an District Center Hospital of Shanghai, Fudan University, Shanghai, China

## Abstract

**Background:**

To evaluate the association between high-sensitivity C-reactive protein (hsCRP) and erythrocyte sedimentation rate (ESR), and diabetic kidney disease (DKD) in patients with type 2 diabetes mellitus (T2DM).

**Methods:**

A cross-sectional study was conducted in 1210 patients with T2DM, among whom 265 had DKD. The severity of DKD was assessed by estimated-glomerular filtration rate (eGFR) and urinary albumin creatinine ratio (ACR). The relationship between ESR, hsCRP and DKD was analyzed by multivariate logistic analysis. The relationship between ESR and eGFR, ESR or ACR was analyzed by multivariate linear regression.

**Results:**

ESR (23.0 [12.0 ~ 41.5] mm/h versus 12.0 [7.0 ~ 22.0] mm/h, *P* <  0.001) and hsCRP (3.60 [2.20 ~ 7.65] versus 2.90 [1.80 ~ 5.60] mg/L mg/L, *P* <  0.01) values were significantly higher in patients with DKD than those without. Patients with higher ESR or hsCRP had lower eGFR and higher ACR. After adjusted for gender, age, hemoglobin, plasma proteins, HbA_1c_, lipid profiles, and the usage of renin-angiotensin system inhibitors, ESR but not hsCRP was independently associated with the rate and severity of DKD in patients with T2DM.

**Conclusion:**

ESR was independently associated with the rate and severity of DKD in patients with T2DM.

## Background

Type 2 diabetic mellitus (T2DM) is a chronic metabolic disorder with multiple complications, including diabetic retinopathy, diabetic neuropathy, diabetic kidney disease (DKD) as well as cardiovascular diseases [1]. DKD affects 20–40% of patients with T2DM, and is the leading cause of end-stage renal disease (ESRD) [2]. With the rapidly growing prevalence of DKD, there is an overwhelming requirement for biomarkers which can predict the omset and severity of DKD.

T2DM is related to an exacerbated systematic inflammation [3]. Chronic inflammation in patients with T2DM is involved in the onset and development of DKD [4]. Mounting evidences have shown that a number of molecules related to inflammation can be predictable in DKD. Urinary tumor necrosis factor-α (TNF-α), interleukin-8 (IL-8), and monocyte chemo-attractant protein-1 (MCP1) are found to be elevated in patients with DKD [5, 6]. Gohda et al. found that circulating TNF receptors were strongly associated with renal function loss in patients with DKD [7]. On ground of this, circulating inflammatory markers might be relevant to the diagnosis and prognosis of DKD [8].

Among all plasma inflammatory biomarkers, erythrocyte sedimentation rate (ESR) and high-sensitivity C-reactive protein (hsCRP) are the mostly commonly used laboratory tests for identifying systematic inflammation [9]. Both ESR and hsCRP are important markers in various inflammation-related diseases. For example, ESR and hsCRP are higher in sarcoidosis patients or osteoarthritis compared to healthy controls [10, 11]. Latest report also revealed that elevated ESR and CRP were associated with the increased urinary albumin excretion [12]. However, there is no study exploring the relationship between ESR, hsCRP and the risk as well as the severity of DKD.

In this study, we retrospectively studied a cohort of 1210 patients with T2DM to investigate the potential relationship between DKD and the degree of systemic inflammation measured by ESR and hsCRP.

## Methods

### Study design and participants

From January 2013 to October 2017, patients with T2DM who were hospitalized in department of Endocrinology and Metabolism of local hospital were enrolled in this study. The study was approved by the ethics committee of Huashan Hospital (Approval No:2014–250). Written informed consent was obtained from all participants. The exclusion criteria were as follows: (1) patients with immune deficiency; (2) patients with a history of operation or acute coronary syndrome within a month; (3) patients with malignant tumors; (4) patients with a history of inflammatory conditions including current infection, rheumatoid arthritis, systemic lupus erythematosus, ankylosing spondylitis, live cirrhosis, tuberculosis, etc.; and (5) patients with the history of steroid usage. All participants included in our study had physical examination and medical history review.

### The criteria for diabetic kidney disease

DKD was defined as patients with macro-albuminuria or patients with micro-albuminuria in the presence of diabetic retinopathy according to the diagnostic criteria from KDOQI clinical practice guidelines [13]. Macro-albuminuria was defined as an albumin creatinine ratio (ACR) > 300 mg/g and micro-albuminuria is defined as ACR between 30 and 300 mg/g in two of three urine sample collections [13].

### Laboratory parameters

Demographic data (age, gender, status of hypertension) were collected from medical records. Hypertension was diagnosed if patients had history of hypertension or had blood pressure above 140/90 mmHg for twice obtained on ≥2 occasions [14]. Fasting blood sample was collected to measure fasting plasma glucose (FBG), glycated hemoglobin (HbA_1c_), hsCRP, serum albumin (ALB), globulin (GLB), total cholesterol (TC), triglyceride (TG), high-density lipoprotein cholesterol (HDL) and low-density lipoprotein cholesterol (LDL), serum creatinine (SCr), and ESR. Besides, the record of renin-angiotensin system (RAS) inhibitors usage was also collected.

The level of HbA_1c_ was measured by liquid chromatography VARIANTTM II and D-10 Systems, BIORAD, USA). ESR was measured using ESR-30 fully automatic dynamic analyzer (Shanghai Xunda Medical Instrument Co., Ltd., China). FBG, TC, TG, HDL, LDL, ALB, total protein, SCr was quantified by Beckman AU5800 (Beckman Coulter Inc., Brea, CA). HsCRP was detected by i-CHROMA reader (Boditech Med inc, Gangwon-do, Korea). Urine was collected for third times and then ACR was immediately measured using Turbidimetry Hitachi system (Roche, Mannheim, Germany). Estimated-glomerular filtration rate (eGFR) was calculated according to CKD-EPI formula (Male: 141 x min (SCr/0.9,1) -0.411 x max (SCr/0.9,1) -1.209 × 0.993 Age; Female: 141 x min (SCr/0.7,1) -0.329 x max (SCr/0.7,1) -1.209 × 0.993 Age× 1.018) [13].

### Statistical analysis

All analyses were performed by SPSS version 21.0 for windows system. Categorical variables were exhibited by frequencies and percentages, with X^2^ test or Fisher’s exact test for detecting the difference. Continuous data was expressed as median values and 25th–75th percentiles because of non-normal distribution analyzed by Kolmogorov-Smirnov test. Kruskal-Wallis test and Mann-Whitney U test were conducted to evaluate the difference. Multivariable linear regression analysis and logistic analysis were used to assess the relationship between inflammatory biomarkers and DKD. A two-tailed *P* <  0.05 was defined as statistically significant.

## Results

### Basic characteristics

A total of 1210 patients with T2DM were included in the current analysis, of whom 265 had DKD. Comparison of the characteristics were listed in Table 1. Compared with patients without DKD, patients with DKD showed higher ESR value (12.0 [7.0 ~ 22.0] mm/h vs 23.0 [12.0 ~ 41.5] mm/h, *P* <  0.001) and higher hsCR*P* value (2.90 [1.80 ~ 5.60] mg/L vs 3.60 [2.20 ~ 7.65] mg/L, *P* <  0.01).

**Table 1:** Characteristics of patients with or without diabetic kidney disease

Variables	Patients without DKD (*n* = 945)	Patients with DKD (*n* = 265)	*P* value
Age (years)	65.24 (59.00 ~ 75.03)	67.00 (59.70 ~ 79.00)	0.005
Female (n, %)	400 (42.33%)	110 (41.51%)	0.83
Smoking (n, %)	275 (29.1%)	76 (28.7%)	0.90
Duration of T2DM (years)	9.80 (5.35 ~ 14.25)	9.10 (5.50 ~ 14.20)	0.71
BMI (kg/m^2^)	25.00 (22.25 ~ 27.85)	24.9 (21.90 ~ 28.10)	0.74
Hypertension (%)	598 (63.28%)	221 (83.40%)	< 0.001
Medicine used (n, %)			
Metformin	502 (53.1%)	107 (40.4%)	< 0.001
Thiazolidinediones	35 (3.7%)	11 (4.2%)	0.74
Statins	402 (42.5%)	123 (46.4%)	0.26
Aspirin	236 (25.0%)	85 (32.1%)	0.021
RASi	365 (40.7%)	151 (57.0%)	< 0.001
ESR (mm/h)	12.00 (7.00 ~ 22.00)	23.00 (12.00 ~ 41.50)	< 0.001
hsCRP (mg/L)	2.90 (1.80 ~ 5.60)	3.60 (2.20 ~ 7.65)	< 0.001
WBC (× 10^9^/L)	6.43 (5.35 ~ 7.76)	6.66 (5.60 ~ 8.01)	0.039
NEU (%)	58.35 (52.03 ~ 64.60)	61.50 (55.45 ~ 68.80)	< 0.001
Hb (g/L)	134.00 (123.00 ~ 146.00)	127.00 (114.00 ~ 136.50)	< 0.001
ALB(g/L)	37.00 (35.00 ~ 40.00)	35.00 (31.00 ~ 38.00)	< 0.001
GLB(g/L)	27.00 (24.00 ~ 30.00)	28.00 (25.00 ~ 31.00)	< 0.001
HbA_1_c (%)	8.20 (7.00 ~ 9.90)	8.60 (7.30 ~ 10.10)	0.10
FBG (mmol/L)	7.26 (5.82 ~ 9.67)	7.90 (6.10 ~ 10.93)	0.017
TC (mmol/L)	4.26 (3.57 ~ 5.07)	4.46 (3.73 ~ 5.39)	0.006
TG (mmol/L)	1.33 (0.92 ~ 1.97)	1.52 (1.01 ~ 2.22)	0.002
HDL (mmol/L)	0.97 (0.84 ~ 1.15)	0.92 (0.80 ~ 1.08)	< 0.001
LDL (mmol/L)	2.38 (1.82 ~ 3.03)	2.60 (1.83 ~ 3.29)	0.005
ACR (mg/g)	12.56 (7.04 ~ 27.38)	394.49 (96.64 ~ 1316.90)	< 0.001
SCr (μmol/L)	67.84 (56.00 ~ 79.93)	84.00 (64.50 ~ 108.40)	< 0.001
eGFR (ml/min/1.73m^2^)	92.04 (78.10 ~ 101.72)	75.69 (48.13 ~ 95.18)	< 0.001

Besides, compared with patients without DKD, those with DKD has higher ACR (12.56 [7.04 ~ 27.38] mg/g vs 394.49 [96.64 ~ 1316.90]mg/g, *P* <  0.001) and lower eGFR (92.04 [78.10 ~ 101.72] ml/min/1.73m^2^ vs 75.69 [48.13 ~ 95.18] ml/min/1.73m^2^, P <  0.001), as well as higher GLB, TC, TG, LDL and lower HDL, ALB levels (Table 1). The use of aspirin (*p* = 0.021) and metformin (*p* <  0.001) between two groups also reached statistical significance.

### Both ESR and hsCRP were associated with renal damage in T2DM

**Fig. 1:**
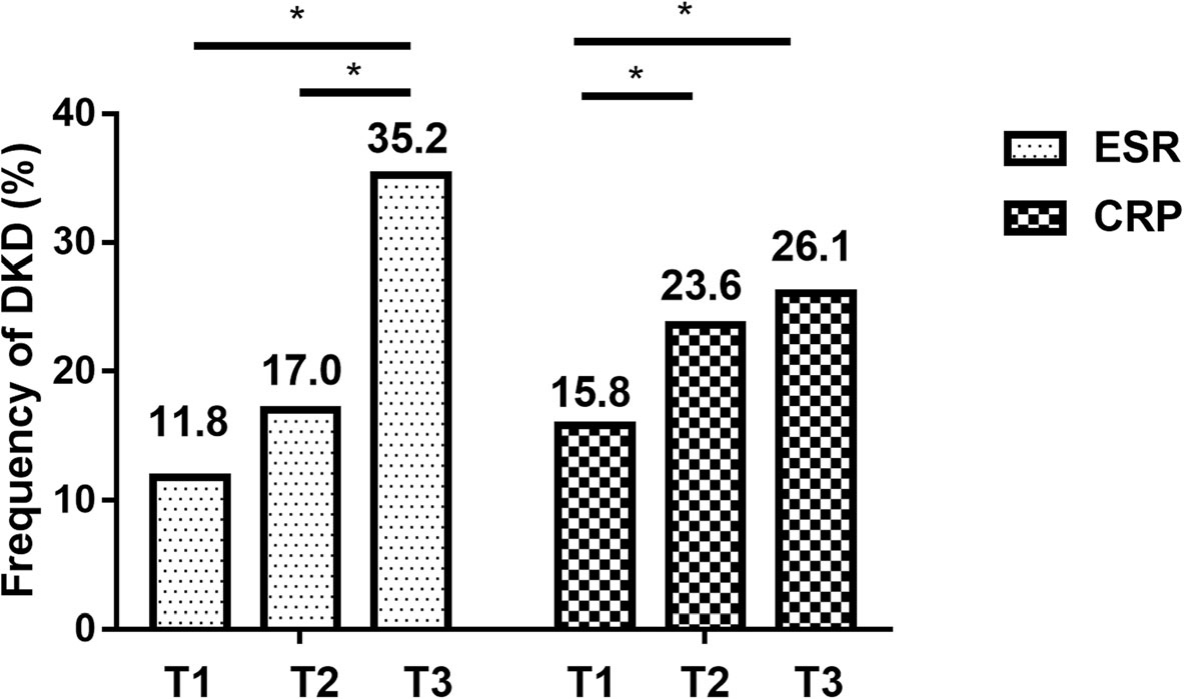
Frequency of DKD in subgroups according to ESR and CRP tertiles. Legends: *: *p* <  0.05. ESR: erythrocyte sedimentation rate, CRP: C reactive protein

Patients were then divided into three subgroups based on the tertiles of ESR (ESR-T1: ≤8 mm/h, ESR-T2: 8 ~ 21 mm/h, and ESR-T3: > 21 mm/h). The occurrence of DKD rose in accompany with ESR elevation (Fig. 1a, ESR-T1: 11.8%, ESR-T2: 17.0%, and ESR-T3: 35.2%, respectively). Specifically, the difference of incidence between ESR-T1 and ESR-T3 (*p* <  0.05), and between ESR-T2 and ESR-T3 (p <  0.05), reached statistical significance. Similarly, by dividing patients into three subgroups based on the tertiles of hsCRP (hsCRP-T1: ≤2.3 mg/L, hsCRP-T2: 2.3 ~ 4.5 mg/L, and hsCRP-T3: > 4.5 mg/L), we found that the occurrence of DKD rose when hsCRP elevated (Fig. 1b, hsCRP-T1: 15.8%, hsCRP-T2: 23.6%, and hsCRP-T3: 26.1%, respectively). The difference of incidence between hsCRP-T1 and hsCRP-T2 (*p* <  0.05), and between hsCRP-T1 and hsCRP-T3 (p <  0.05), reached significance.

**Fig. 2:**
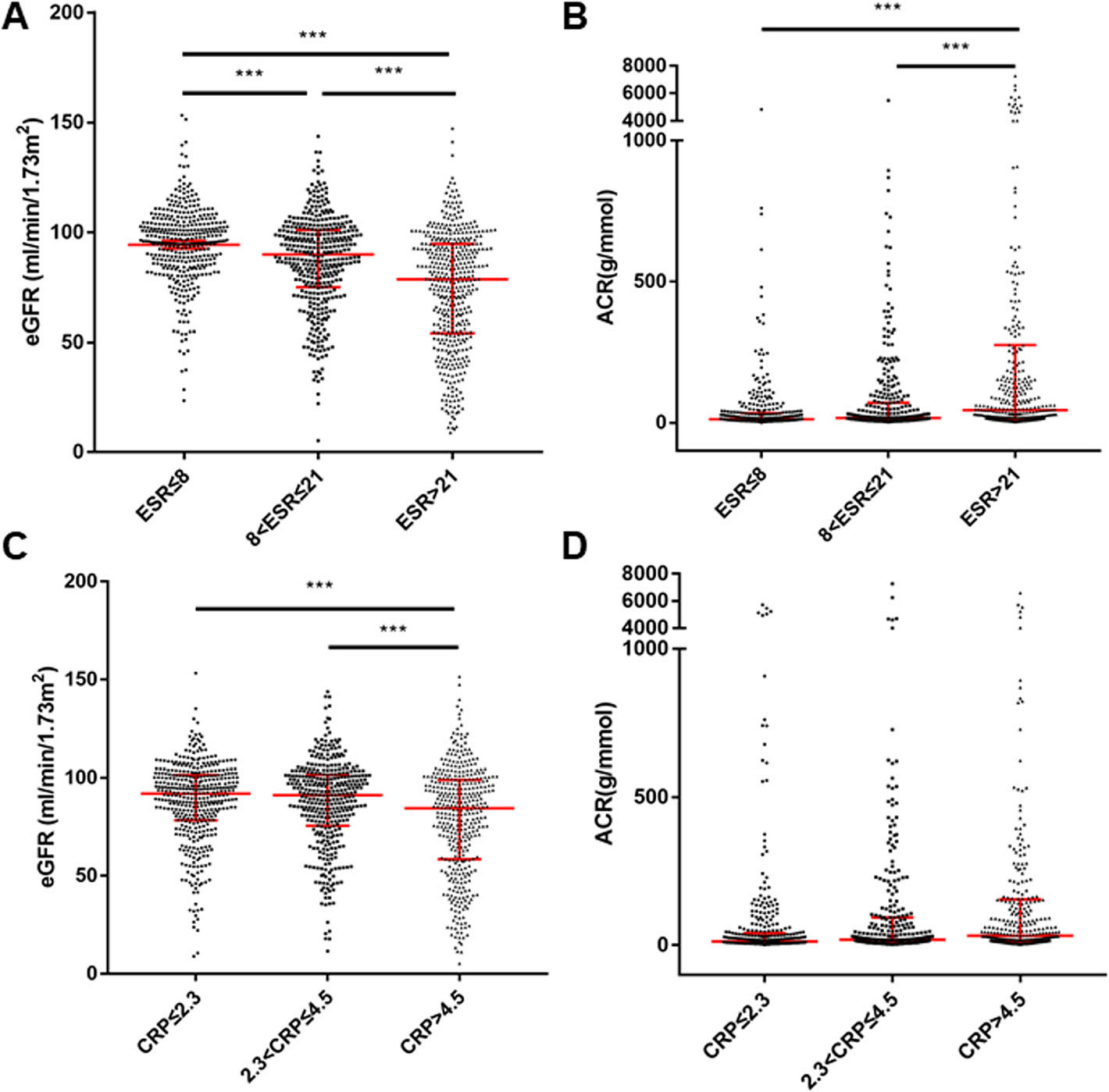
eGFR and ACR according to ESR or CRP tertiles. Data was shown as median with interquartiles. ***: *p* <  0.001. ESR: erythrocyte sedimentation rate, CRP: C reactive protein, eGFR: estimated glomerular filtration rate, ACR: albumin creatinine ratio

Next, we examined the value of eGFR and ACR according to the tertiles of ESR or hsCRP. We found that from ESR-T1 to ESR-T2, and to ESR-T3, eGFR decreased from 95.74 (85.17 ~ 104.37) ml/min/1.73m^2^ to 89.95 (74.96 ~ 101.12) ml/min/1.73m^2^, and to 78.61 (54.09 ~ 94.77) ml/min/1.73m^2^ (Fig. 2a), while ACR increased from 12.42 (6.61 ~ 33.83) mg/g to 16.64 (7.68 ~ 70.54) mg/g, and to 44.08 (12.42 ~ 275.32) mg/g (Fig. 2b). Similarly, from hsCRP-T1 to hsCRP-T2, and to hsCRP-T3, eGFR decreased from 91.94 (78.41 ~ 101.34) ml/min/1.73m^2^ to 91.17 (75.62 ~ 101.55) ml/min/1.73m^2^, and to 84.49 (58.48 ~ 98.92) ml/min/1.73m^2^ (Fig. 2c), while ACR increased from 12.61 (7.33 ~ 40.99) mg/g to 18.61 (7.94 ~ 93.59) mg/g, and to 32.12 (11.80 ~ 155.08) mg/g (Fig. 2d).

**Fig. 3:**
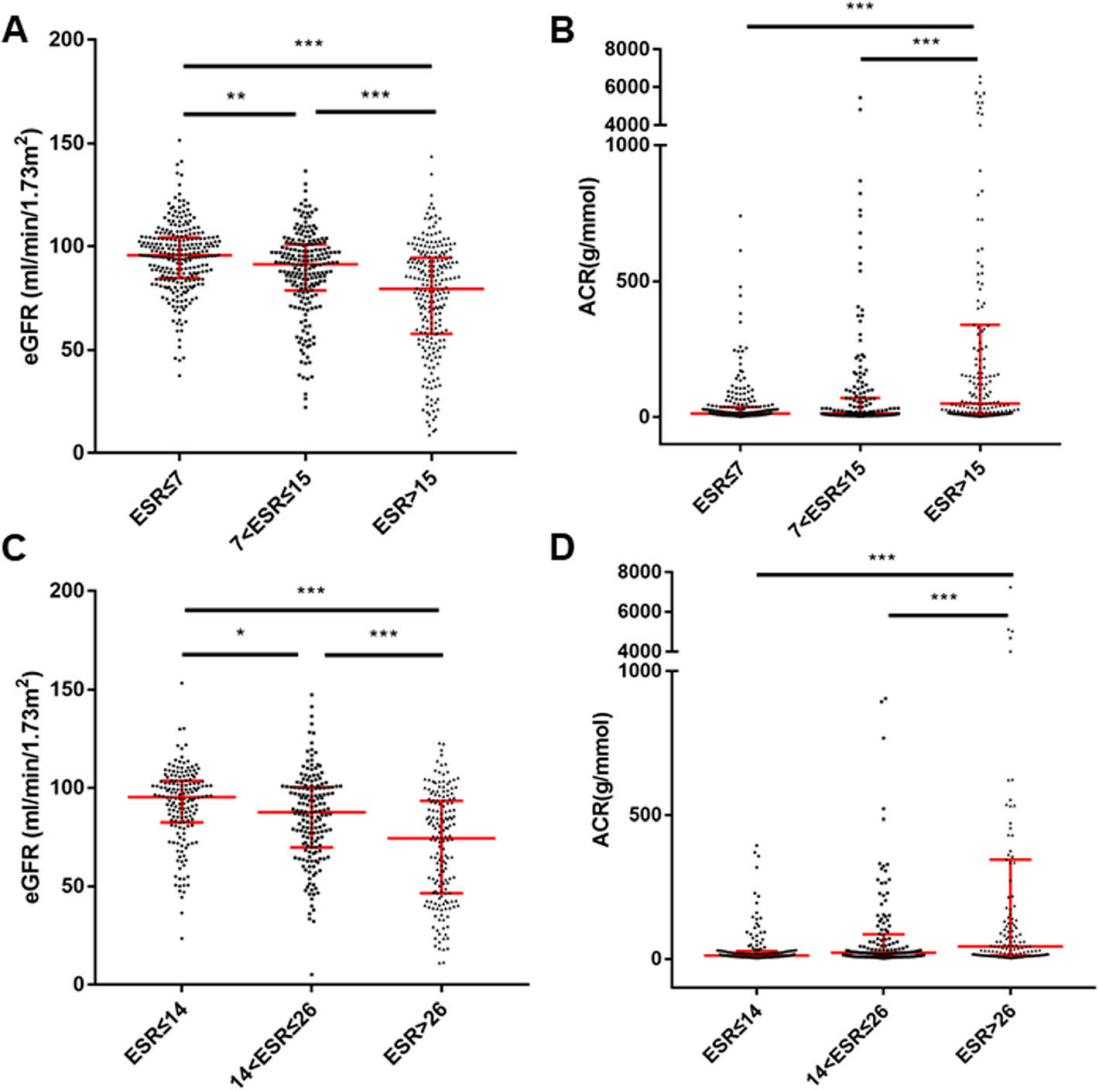
eGFR and ACR according to ESR tertiles in female and male. Data was shown as median with interquartiles. *: *p* <  0.05, **: *p* <  0.01, ***: *P* <  0.001. ESR: erythrocyte sedimentation rate, eGFR: estimated glomerular filtration rate, ACR: albumin creatinine ratio

Given the different ESR normal range between male and female, gender-based subgroup analysis was conducted. Based on subgroup data, the tertiles for male were ≤ 7 mm/h (mT1), 7 ~ 15 mm/h (mT2), and > 15 mm/h (mT3). Accordingly, eGFR decreased from 95.82 (84.75 ~ 104.31) to 91.41 (78.85 ~ 100.92), and to 79.62 (57.82 ~ 94.56) (Fig. 3a), while ACR increased from 12.84 (6.59 ~ 37.00) to 14.38 (6.59 ~ 70.76), and to 49.56 (12.29 ~ 340.07) (Fig. 3b).

For female, the tertiles were ≤ 14 mm/h (fT1), 14 ~ 26 mm/h (fT2), and > 26 mm/h (fT3). Accordingly, eGFR decreased from 95.24 (82.57 ~ 103.50) to 87.59 (69.89 ~ 100.35) (Fig. 3c), and to 74.52 (46.51 ~ 93.40), while ACR increased from 12.33 (7.36 ~ 28.05) to 22.00 (8.41 ~ 86.10), and to 43.85 (13.35 ~ 345.77) (Fig. 3d).

### ESR was independently associated with DKD in T2DM

Logistic analysis showed that the risk of DKD increased with the upregulation of ESR, even after adjustment for age, gender, hypertension, hemoglobin, TC, HDL, ALB, GLB, HbA_1c_, and the usage of RAS inhibitor. However, hsCRP was not an independent risk factor for DKD after adjustment for confounders (Table 2). Furthermore, based on the adjusted model, ESR was significantly negatively related to eGFR and positively correlated to ACR (Table 3).

**Table 2:** Relationship between erythrocyte sedimentation rate or high-sensitivity C-reactive protein levels and diabetic kidney disease

Variables	Group	Model 1 OR (95% CI)	*P* value	Model 2 OR (95% CI)	*P* value
ESR (mm/h)	T1 (< 9)	reference		reference	
	T2 (9 ~ 20)	1.52 (1.01 ~ 2.29)	0.04	1.22 (0.78 ~ 1.93)	0.39
	T3 (≥20)	4.04 (2.80 ~ 5.84)	< 0.001	2.42 (1.45 ~ 4.03)	< 0.001
hsCRP (mg/L)	T1 (< 2.2)	reference		reference	
	T2 (2.2 ~ 4.5)	1.65 (1.15 ~ 2.35)	0.006	1.05 (0.68 ~ 1.61)	0.83
	T3 (≥4.5)	1.89 (1.34 ~ 2.67)	< 0.001	1.40 (0.95 ~ 2.08)	0.09

Model 1: unadjusted,

Model 2: adjusted for age, gender, hypertension, hemoglobin, TC, HDL, ALB, GLB, HbA_1c_, and the usage of RAS inhibitor, metformin, and aspirin

**Table 3:** Multivariate association of erythrocyte sedimentation rate and severity of diabetic kidney disease

Clinical parameters	ESR			
	B	SE	beta	P
eGFR	−0.262	0.031	−0.137	< .001
ACR	6.040	1.486	0.155	< .001

## Discussion

In this study, we found that patients with DKD had higher ESR and hsCRP levels than those without DKD. Moreover, ESR but not hsCRP was independently related to the risk and severity of DKD, as indicated by both eGFR and ACR.

Growing evidences underline the critical role of inflammation in the progression of DKD. In the early stage of DKD, macrophages accumulate in kidney and produce cell adhesion molecules, chemokines, and pro-inflammatory cytokines [15, 16], which recruit more macrophages into kidney and exacerbate inflammatory injury [17].

Inflammatory parameters such as TNF-α have been reported to be correlated with renal function in T2DM [18–22], suggesting the predictive potential of inflammatory marker in this disorder [23]. Numerous factors have been found to be prognostic. For example, Hussain et al. found that galectin-3 and growth differentiation factor-15 were inversely related to eGFR and could be used as a biomarker of renal function [24]. Bian et al. identified serum Activin A as an indicator for the treatment efficacy of DKD [25]. By reviewing the progression of DKD, Cao et al. summarized several microRNAs that could be used as biomarkers and therapeutic targets in DKD [26]. However, the measurement of these factors is expensive, which limits their clinical application. On the other hand, ESR and hsCRP can provide valuable information in terms of inflammatory status in a cheap and convenient manner [27]. They are influenced by various inflammatory factors, making them sensitive to inflammation [28]. In the present study, we found that ESR, instead of hsCRP, was independently associated with the incidence and severity of DKD, indicating the role of ESR for prognosticating DKD onset and progression.

ESR has been in use since 1921 as a test of inflammatory reaction for tuberculosis [27]. As an indicator of inflammation, ESR is widely used as a predictive biomarker in various chronic diseases, including anti-neutrophil cytoplasmic antibody-associated vasculitis [29] and systematic inflammatory response syndrome [30], and can be an independent prognostic factor for osteomyelitis recurrence in patients with T2DM [31]. However, the relationship between ESR and DKD has not been proposed. Based on our findings, ESR elevated significantly in the patients with DKD and is independently associated with DKD. According to our multivariate logistic analysis, patients with higher ESR level were more likely to have concomitant DKD than those with lower ESR. Furthermore, ESR was positively related to the severity of DKD, measured by both renal function and urinary albumin secretion, suggesting the correlation between inflammation and DKD progression. Above all, as a widely-applied and inexpensive measurement, ESR can be an ideal parameter for DKD occurrence and severity in patients with T2DM.

Despite of few literatures on ESR and DKD, the relationship between hsCRP and T2DM or DKD has been widely investigated. In a cross-sectional study including 64 patients with T2DM, hsCRP was significantly higher in those with micro-albuminuria compared to those with normo-albuminuria [32]. Another study reported that hsCRP rose significantly in patients with CKD than those without CKD [33]. However, whether CRP is an independent risk factor for DKD is still controversial. Navarro et al. found that hsCRP was independently associated with albuminuria in T2DM [34]. By contrast, in a study with 467 patients with diabetes and 1014 controls, hsCRP was not independently related to micro-albuminuria [35]. Similarly, a research showed that hsCRP was not independently related to eGFR or urinary albumin secretion, after adjusted for CKD risk factors [33]. Based on our findings with a sample size of 1210, hsCRP was not independently associated with DKD after adjusted confounding factors, although the hsCRP value was significantly higher in patients with DKD than those without DKD.

The current outcomes should still be interpreted with caution. First, this is a cross-sectional study which might have introduced bias. Longitudinal studies are required to define whether ESR could predict the onset of DKD. Besides, ESR was only obtained from a single measurement, which might not be able to reflect a relation over time. However, after taking confounders into consideration, ESR are still associated with measures of DKD.

## Conclusion

In conclusion, the current research underlined the role of inflammation in DKD. Our study found that, both ESR and hsCRP correlated with DKD in T2DM, of which ESR was an independent risk factor for DKD and positively associated with severity of DKD.

## Data Availability

The datasets used and/or analyzed during the current study are available from the corresponding author on reasonable request.

## Abbreviations

hsCRP: High-sensitivity C-reactive protein
ESR: Erythrocyte sedimentation rate
DKD: Diabetic kidney disease
T2DM: Type 2 diabetes mellitus
eGFR: Estimated-glomerular filtration rate
ACR: Albumin creatinine ratio
ESRD: End-stage renal disease
TNF-α: Tumor necrosis factor-α
IL-8: Interleukin-8
MCP1: Monocyte chemo-attractant protein-1
FBG: Fasting plasma glucose
HbA_1c_: Glycated hemoglobin
ALB: Albumin
GLB: Globulin
TC: Total cholesterol
TG: Triglyceride
HDL: High-density lipoprotein cholesterol
LDL: Low-density lipoprotein cholesterol
SCr: Serum creatinine
RAS: Renin-angiotensin system
RASi: Renin-angiotensin system inhibitor
WBC: White blood cell
NEU: Neutrophil granulocyte
Hb: Hemoglobin
BMI: Body mass index
OR: Odds ratio
CI: Confidence interval
T: Tertile
SE: Standard error
